# Sedation Agents Differentially Modulate Cortical and Subcortical Blood Oxygenation: Evidence from Ultra-High Field MRI at 17.2 T

**DOI:** 10.1371/journal.pone.0100323

**Published:** 2014-07-22

**Authors:** Lynn Uhrig, Luisa Ciobanu, Boucif Djemai, Denis Le Bihan, Béchir Jarraya

**Affiliations:** 1 NeuroSpin, Commissariat à l'Energie Atomique et aux Energies Alternatives, CEA Saclay, Gif-sur-Yvette, France; 2 Equipe Avenir INSERM Bettencourt Schueller, NeuroSpin, Unité de Recherche en NeuroImagerie Applicative Clinique et Translationnelle (UNIACT), Gif-sur-Yvette, France; 3 Department of Neurosurgery, Neuromodulation Unit, Foch Hospital, University of Versailles Saint-Quentin, Suresnes, France; Penn State University, United States of America

## Abstract

**Background:**

Sedation agents affect brain hemodynamic and metabolism leading to specific modifications of the cerebral blood oxygenation level. We previously demonstrated that ultra-high field (UHF) MRI detects changes in cortical blood oxygenation following the administration of sedation drugs commonly used in animal research. Here we applied the UHF-MRI method to study clinically relevant sedation drugs for their effects on cortical and subcortical (thalamus, striatum) oxygenation levels.

**Methods:**

We acquired T2*-weighted images of Sprague-Dawley rat brains at 17.2T *in vivo*. During each MRI session, rats were first anesthetized with isoflurane, then with a second sedative agent (sevoflurane, propofol, midazolam, medetomidine or ketamine-xylazine) after stopping isoflurane. We computed a T2*-oxygenation-ratio that aimed at estimating cerebral blood oxygenation level for each sedative agent in each region of interest: cortex, hippocampus, thalamus and striatum.

**Results:**

The T2*-oxygenation-ratio was consistent across scan sessions. This ratio was higher with inhalational agents than with intravenous agents. Under sevoflurane and medetomidine, T2*-oxygenation-ratio was homogenous across the brain regions. Intravenous agents (except medetomidine) induced a T2*-oxygenation-ratio imbalance between cortex and subcortical regions: T2*-oxygenation-ratio was higher in the cortex than the subcortical areas under ketamine-xylazine; T2*-oxygenation-ratio was higher in subcortical regions than in the cortex under propofol or midazolam.

**Conclusion:**

Preclinical UHF MRI is a powerful method to monitor the changes in cerebral blood oxygenation level induced by sedative agents across brain structures. This approach also allows for a classification of sedative agents based on their differential effects on cerebral blood oxygenation level.

## Introduction

Anesthetic agents directly affect cerebral blood flow (CBF) [1] and metabolism [2], leading to specific modifications of the cerebral blood oxygenation (CBO) level. The investigation of these brain effects is key to optimize the safety and efficacy of anesthetics, and to understand their mechanisms of action. However the available methods for measuring CBO level suffer from invasiveness (continuous jugular venous oxygen saturation measurement [3], use of ionizing radiation [4]), lack of whole brain coverage (cerebral near infrared spectroscopy [5]) or sensitivity [6]. Magnetic resonance imaging (MRI), by using the appropriate pulse sequences, can be sensitized to subtle changes in cerebral blood volume [7], perfusion [8] and oxygenation [9], [10]. Because of its paramagnetic properties in venous blood, deoxyhemoglobin acts as an endogenous contrast agent for MRI T2* sequences, allowing for a noninvasive measurement of CBO level [11]–[13]. Because the effects of anesthetic agents on blood oxygenation level can be subtle, they are challenging to detect with T2*MRI [6]. The emergence of ultra-high field magnetic resonance imaging (UHF-MRI) technology paved the way to discover new neuroimaging contrasts. We recently used UHF-MRI to investigate the effects of common anesthetics in the experimental research field on the rat CBO levels in cortical structures [6] and could demonstrate that UHF-MRI at 17.2T provides a significantly higher sensitivity to monitor *in vivo* the effects of the anesthetic agents on CBO level, than high field MRI at 7T. Having demonstrated the proof of concept [6], here we applied UHF-MRI to study the brain effects of anesthetic agents that are widely used in the clinical practice and in research studies [2], [14], [15]. We demonstrate that UHF-MRI T2* is a reliable biomarker that reflects cerebral blood oxygenation variations among anesthetic agents, offering a classification of these agents based on their thalamo-cortical blood oxygenation.

## Materials and Methods

### Animals

Sprague-Dawley male rats (n = 21, 200–400 g, Janvier, France) were included. All animal studies were conducted in accordance with the European convention for animal care. The protocol was approved by the Committee on the Ethics of Animal Experiments of the CEA (Comité d'Ethique en Expérimentation Animale, CETEA, Permit Number: ID 10_032).

#### General anesthesia protocol

We applied the same experimental set-up described previously [6]. To ensure for the robustness of the anesthesia-MRI experimental procedure and MRI quantitative measures across different sessions, we systematically scanned the animals under isoflurane in the beginning of each session, whatever the studied anesthetic agent. Thus we could compare quantitative MR indices for a same drug (i.e. isoflurane) between sessions.

All animals were first anesthetized with isoflurane (≈1.2 minimum alveolar concentration (MAC)) and a set of gradient echo images was acquired. Then, once the MRI acquisition under isoflurane was performed, isoflurane was stopped and the animal was subsequently anesthetized with one of the following agents: propofol group (n = 5, loading dose of 7.5 mgkg^−1^ IV followed by a continuous IV infusion of 45 mgkg^−1^ h^−2^) [16], midazolam group (n = 5, loading dose of 2 mgkg^−1^, followed by a continuous IV infusion of 0.25 mgkg^−1^ min^−2^) [17], sevoflurane group (n = 5, ≈2.4MAC), medetomidine group (n = 3, bolus of 0.3 mgkg^−1^ IV), or ketamine-xylazine group (n = 3, bolus of 100 mg10 kg^−1^ IP). The second set of gradient echo images was acquired under the second anesthetic agent 30 minutes after isoflurane was stopped and the second anesthetic agent was started. The level of anesthesia was clinically determined by the loss of righting reflex in the rat. All animals were orally intubated and ventilated (fraction of inspired oxygen 0.33; tidal volume 10 mlkg^−1^, respiration rate: 60 min^−1^ adjusted to ensure normocapnic and normoxic conditions).

All monitored physiological parameters (respiration rate, O_2_ saturation, expired CO_2_, temperature) were kept constant through the MRI experiment. Arterial blood gases (pH, paO2, paCO2) were sampled after the intubation and immediately at the end of the MRI acquisition and analyzed using a blood gas analyzer (Radiometer, Copenhagen). Because of the lack of MR compatible technology for blood pressure monitoring at 17.2T, blood pressure could not be monitored during MRI session. However, we checked for the blood pressure stability of all our anesthesia protocols outside the scanner [6]. Furthermore, we performed an invasive monitoring of blood pressure in five rats, i.e. in one representative of each group (three rats in the caudal arteria and two rats in the femoral arteria (SAII, StonyBrook, USA)). At the end of each anesthesia MRI experiement, the rat woke up and moved freely.

### MRI acquisitions

The experiments were performed on a 17.2 T horizontal bore MRI scanner (BioSpec, Bruker BioSpin, Etlingen, Germany). A 3 cm diameter transmit/receive coil (Bruker BioSpin, Etlingen, Germany) was used. Coronal T2* gradient echo and SWI (susceptibility weighted imaging) images were acquired in magnitude and phase, after scout scans and magnetic field homogeneity optimization (FASTMAP). Sequence parameters were: 2D acquisition, in-plane resolution = 80 µm, FOV = 25.6 mm (matrix size 320×320), flip angle α = 45°, TR/TE = 350/8 ms, thickness = 0.2 mm, number of slices = 16, NEX = 14.

#### Data processing

For each T2* gradient echo magnitude image, we manually segmented four regions of interest (cortex, hippocampus, striatum, thalamus) on coronal sections, based on the Paxinos atlas [18].

**Figure 1:**
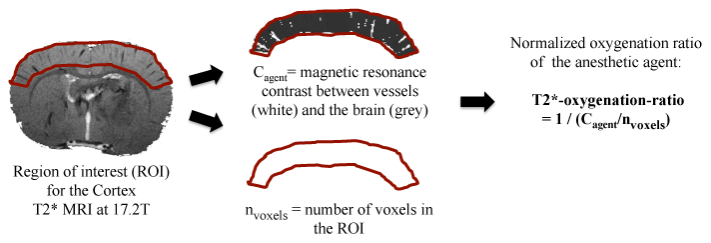
Quantitative method for the estimation of regional cerebral blood oxygenation. T2* magnetic resonance images are acquired at 17.2T in anesthetized rats. Manual segmentation is performed on coronal sections to delineate each region of interest (ROI), here the cortex. For each anesthetic molecule (termed ‘agent’), and for each extracted ROI, the magnetic resonance contrast (C_agent_) between the vessels (hypointense) and brain is automatically computed, reflecting the quantity of deoxyHb of the blood [6]. The number of voxels of the ROI is also calculated. The index we computed, T2*-oxygenation-ratio, is normalized to the number of voxels of the ROI, and reflects the cerebral blood oxygenation for the ROI and the anesthetic agent [6].

For each anesthetic molecule (termed ‘agent’), and for each extracted region of interest (ROI), the relative magnetic resonance contrast (C_agent_) between the vessels (hypointense) and brain is computed, reflecting the quantity of deoxyhemoglobin of the blood (Figure 1) [6]: C_agent_ was measured as the number of voxels with hypointense MR signal within vessels (compared to surrounding tissues) for each sedative agent, by extracting the average signal intensity and the number of voxels with intensities smaller than 75% of this average [6], [19], [20]. The intensity threshold of 75% was selected based on the minimal contrast to noise ratio (CNR) of 7 for discrimination of venous voxels, since the average signal to noise ratio (SNR) (measured in regions without prominent blood vessels) was 28±1.7 at a TE of 8 ms [6]. The CNR between tissue and vascular voxels can be determined as a function of this 75% threshold (τ) and the SNR of the images using the relationship: CNR = (1−τ)×SNR. Given the SNR of 28±1.7 for our images, the CNR was around 7, thus exceeding the minimal value required for reasonable discrimination [6], [21]. The number of hypointense voxels was normalized to the total number of voxels of the ROI (n_voxels_). Thus the oxygenation ratio index we computed, T2*-oxygenation-ratio, was normalized to the voxels of the ROI, and reflected the cerebral blood oxygenation for the ROI and the anesthetic agent [6]: T2*-oxygenation-ratio = 1/(C_agent_/n_voxels_) (Figure 1). The data were analyzed using Matlab R2012a (MathWorks, Massachusetts).

### Statistical analysis

We applied nonparametric statistical tests. To compare brain regions data, we performed a related-samples Friedman's two-way analysis of variance by ranks, followed by Dunn-Bonferroni (D-B) post hoc test for pairwise comparisons. To test for the normality of the distribution of isoflurane data across sessions, we performed a one-sample Kolmogorov-Smirnov (K-S) test. To compare T2*-oxygenation-ratio of different anesthetic agents in each brain region, we performed independent-samples Kruskal-Wallis (K-W) test, followed by Mann-Whitney (M-W) post hoc test for pairwise comparisons. Data were analyzed using the IBM SPSS Statistics software (V.21 for MacOSX).

## Results

### Physiologic measurements

During all the MRI experiments the ventilation parameters were adjusted to maintain constant exhaled CO_2_. PaCO_2_ was between 41–46 mmHg, PaO_2_ >200 mmHg, pH:7.30–7.45. Noninvasive measures of PaCO_2_ directly correlate with invasive measures in our preclinical set up [6]. Mean arterial blood pressure was (mean+/−standard deviation): Isoflurane(n = 5): 79.7+/−2.1 mmHg, propofol(n = 1):78.5+/−2.5 mmHg, midazolam(n = 1):72+/−2 mmHg, sevoflurane(n = 1):78.5+/−1 mmHg, medetomidine(n = 1):87.0+/−9.3 mmHg [6] and ketamine/xylazine(n = 1):69.9+/3.6 mmHg [6].

### MR signal across sessions

Because we scanned all the rats with isoflurane (n = 21), T2*-oxygenation-ratio was calculated under isoflurane in the different ROIs and compared for consistency across sessions as a benchmark. There was no significant differences of T2*-oxygenation-ratio between the sessions during which the other anesthetics were studied, whatever the considered ROI: cortex (T2*-oxygenation-ratio = 125690+/−14 129 (mean+/−standard deviation), p = 0.64, K-W test), thalamus (T2*-oxygenation-ratio = 84081+/−4 976 (mean+/−standard deviation), p = 0.82, K-W test), hippocampus (T2*-oxygenation-ratio = 124671+/−8 901 (mean+/−standard deviation), p = 0.45, K-W test), striatum (T2*-oxygenation-ratio = 132574+/−12 7523 (mean+/−standard deviation), p = 0.75, K-W test).

### Reliability of T2*-oxygenation-ratio

In this study we introduced a new normalized index, T2*-oxygenation-ratio, in order to estimate CBO in each specified brain region under each tested anesthetic agent. Having demonstrated the consistency of this index across the different MRI scanning sessions under isoflurane, we checked for its normality and found that, whatever the considered brain region, our T2*-oxygenation-ratio followed a normal distribution (K-S test, p = 0.94 for the cortex, p = 0.91 for the hippocampus, p = 0.55 for the thalamus, p = 0.34 for the striatum) (Figure S1). Thus, T2*-oxygenation-ratio appeared as a reliable quantitative measure across MRI scan sessions and across brain regions at 17.2T.

### Effect of sedative agents on T2* contrast

**Figure 2:**
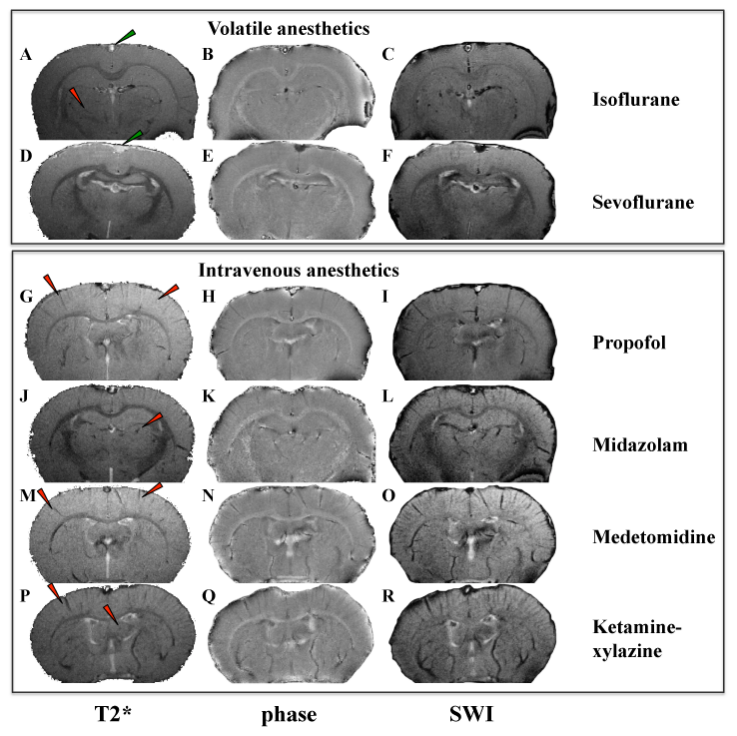
Magnetic resonance images of the rat brain at 17.2T. Coronal sections from images that were acquired under different anesthetic agents. T2* magnitude, left panel; T2* phase, middle panel; susceptibility weighted images (SWI), right panel. Red arrowheads refer to hypointense signal corresponding to brain vessels. Images acquired under volatile anesthetic agents (isoflurane A,B,C; sevoflurane D,E,F) display more homogenous MR signal and less contrast between the brain vessels and the brain parenchyma, than intravenous anesthetics (propofol G,H,I; midazolam J,K,L; medetomidine M,N,O; ketamine-xylazine P,Q,R). Green arrowheads refer to hyperintense signal of the sagittal sinus reflecting increased blood oxygenation with isoflurane and sevoflurane as compared to the other anesthetics.

At 17.2T, magnetic resonance T2* magnitude, T2* phase, and SWI images displayed a striking difference in contrast depending on the anesthetic agent that was used to acquire these images (Figure 2). Compared to intravenous anesthetics, volatile anesthetic agents displayed more homogenous T2* MRI signal with less contrast between brain vessels and parenchyma, and a hyperintense sagittal sinus signal (Figure 2, green arrowheads).

#### Volatile anesthetics

**Figure 3:**
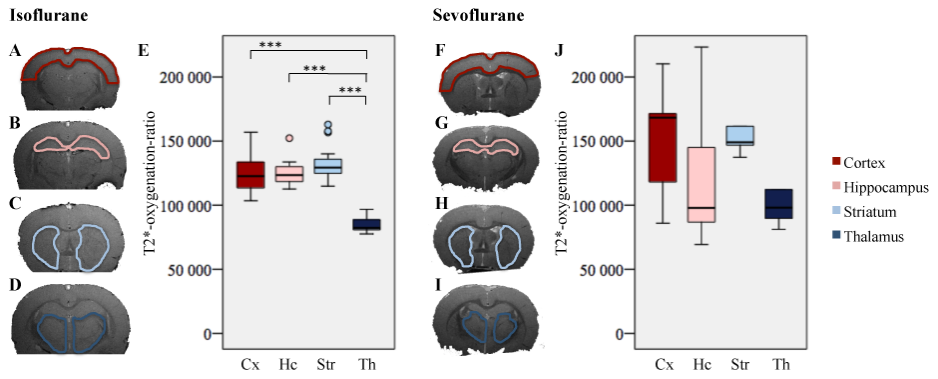
Comparison of T2*-oxygenation-ratio of volatile anesthetics among brain regions. Images are coronal T2* MRI sections acquired *in vivo* under general anesthesia using isoflurane (A: cortex, B: hippocampus, C: striatum, D: thalamus) or sevoflurane (F: cortex, G: hippocampus, H: striatum, I: thalamus). Box plots represent median, 25^th^ and 75^th^ percentile, minimum and maximum values, outliers (°) and extremes (*). Y-axis: regional T2*-oxygenation-ratio. X-axis: Cx, cortex; Hc, hippocampus; Str, stiatum; Th, thalamus. E: T2*-oxygenation-ratio for isoflurane. The thalamus had a lower oxygenation level than the other studied brain regions. J: T2*-oxygenation-ratio for sevoflurane. No significant difference was observed between the studied brain regions. *** p<0.001.

Under isoflurane, T2*-oxygenation-ratio varied across brain regions (Friedman's test, p<0.001) with the thalamus oxygenation ratio being lower than cortex, hippocampus and striatum (D-B post hoc test, p<0.001, Figure 3). No statistical differences were observed between cortex, hippocampus and striatum under isoflurane anesthesia (D-B post hoc test: cortex vs hippocampus, p = 0.81; cortex vs striatum, p = 0.12; hippocampus vs striatum, p = 0.19). There were no significant differences of T2*-oxygenation-ratio across brain regions under sevoflurane (Friedman's test, p = 0.08, Figure 3).

#### Intravenous anesthetics

**Figure 4:**
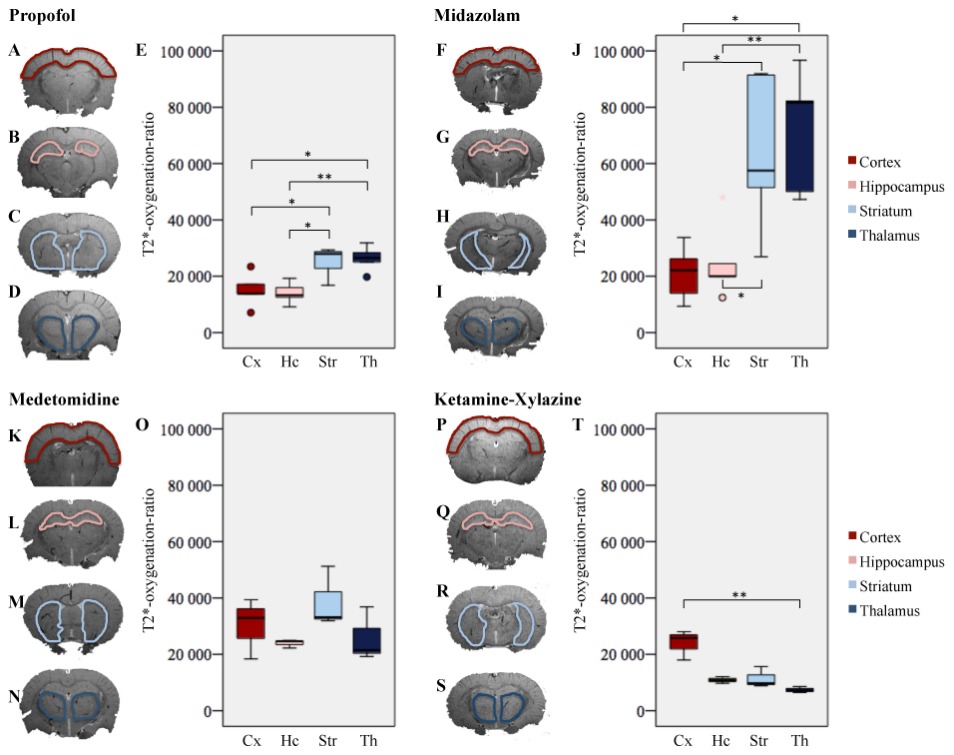
Comparison of T2*-oxygenation-ratio of intravenous anesthetics among brain regions. Images are coronal T2* MRI sections acquired *in vivo* under general anesthesia using propofol (A: cortex, B: hippocampus, C: striatum, D: thalamus), midazolam (F: cortex, G: hippocampus, H: striatum, I: thalamus), medetomidine (K: cortex, L: hippocampus, M: striatum, N: thalamus) or ketamine-xylazine (P: cortex, Q: hippocampus, R: striatum, S: thalamus). Box plots represents median, 25^th^ and 75^th^ percentile, minimum and maximum values, outliers (°) and extremes (*). Y-axis: regional T2*-oxygenation-ratio. X-axis: Cx, cortex; Hc, hippocampus; Str, stiatum; Th, thalamus. E: T2*-oxygenation-ratio for propofol. J: T2*-oxygenation-ratio for midazolam, O: T2*-oxygenation-ratio for medetomidine, T: T2*-oxygenation-ratio for ketamine-xylazine. * p<0.05, ** p<0.01.

The T2*-oxygenation-ratio varied across brain regions under propofol (Friedman's test, p = 0.007), midazolam (Friedman's test, p = 0.007) and ketamine-xylazine (Friedman's test, p = 0.04) but not under medetomidine (Friedman's test, p = 0.33, Figure 4).

Propofol and midazolam had a similar profile: under each drug, the striatum and the thalamus had equivalent T2*-oxygenation-ratio (D-B post hoc test, p = 0.81) but a higher T2*-oxygenation-ratio than the cortex (D-B post hoc test, p = 0.027 and 0.014 respectively) and the hippocampus (D-B post hoc test, p = 0.014 and 0.007 respectively, Figure 4).

Under ketamine-xylazine, T2*-oxygenation-ratio in the cortex was higher than in the thalamus (D-B post hoc test, p = 0.004, Figure 4). No statistical differences were observed between the remaining structures under ketamine-xylazine anesthesia (D-B post hoc test: cortex vs hippocampus, p = 0.20; cortex vs striatum, p = 0.11; hippocampus vs striatum, p = 0.75; hippocampus vs thalamus, p = 0.11; striatum vs thalamus, p = 0.20; Figure 4).

### Differential effects of sedative agents across brain regions

Sedative drugs induced striking differences of T2*-oxygenation-ratio across brain regions, i.e. cortex, hippocampus, striatum and thalamus (Friedman's test, p<0.001).

#### Cortex

**Figure 5:**
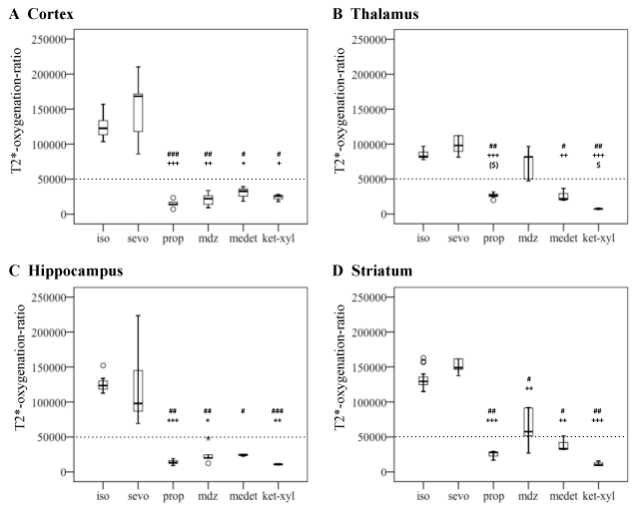
Differences of T2*-oxygenation-ratio between anesthetic agents. A, cortex; B, thalamus; C, hippocampus; D, striatum. Diagrams are box plots made with IBM SPSS software. Each box plot represents median, 25^th^ and 75^th^ percentile, minimum and maximum values, outliers (°) and extremes (*). Y-axis: regional T2*-oxygenation-ratio. Compared to isoflurane: # p<0.05, ## p<0.01, ### p<0.001. Compared to sevoflurane: + p<0.05, ++ p<0.01, +++ p<0.001. Compared to midazolam: $ p<0.05, ($) p = 0.053. iso, isoflurane; sevo, sevoflurane; prop, propofol; mdz, midazolam; medet, medetomidine; ket-xyl, ketamine-xylazine. The dotted line at 50000 shows a separation between high and low T2*-oxygenation-ratio.

Anesthetic agents significantly affected the T2*-oxygenation-ratio of the cortex (K-W test, p<0.001, Figure 5A). Under isoflurane or sevoflurane, cortical T2*-oxygenation-ratio was higher than with propofol (M-W post hoc, p<0.001 in both cases), midazolam (M-W post hoc, p = 0.001 and 0.002 respectively), medetomidine (M-W post hoc, p = 0.035 and 0.023 respectively) and ketamine-xylazine (M-W post hoc, p = 0.015 and 0.011 respectively). No statistical differences were observed between propofol, midazolam, medetomidine and ketamine-zylazine for the T2*-oxygenation-ratio.

#### Thalamus

Anesthetic agents significantly affected the T2*-oxygenation-ratio of the thalamus (K-W test, p<0.001, Figure 5B). Under isoflurane or sevoflurane, thalamic T2*-oxygenation-ratio was higher than with propofol (M-W post hoc, p = 0.003 and p<0.001 respectively), medetomidine (M-W post hoc, p = 0.013 and 0.001 respectively) and ketamine-xylazine (M-W post hoc, p = 0.002 and p<0.001 respectively). Midazolam had higher thalamic T2*-oxygenation-ratio than ketamine-xylazine (M-W post hoc, p = 0.020). The difference between T2*-oxygenation-ratio of the thalamus under propofol and under midazolam approached the level of significance (M-W post hoc, p = 0.053). No statistical differences were observed within the thalamus between the other anesthetics.

#### Hippocampus

Anesthetic agents significantly affected the T2*-oxygenation-ratio of the hippocampus (K-W test, p<0.001, Figure 5C). Isoflurane had higher T2*-oxygenation-ratio than propofol (M-W post hoc, p<0.001), midazolam (M-W post hoc, p = 0.002), medetomidine (M-W post hoc, p = 0.031) and ketamine-xylazine (M-W post hoc, p<0.001). Sevoflurane displayed higher T2*-oxygenation-ratio than propofol (M-W post hoc, p = 0.007), midazolam (M-W post hoc, p = 0.037), and ketamine-xylazine (M-W post hoc, p = 0.007). No statistical differences were observed within the hippocampus between the other anesthetics.

#### Striatum

Anesthetic agents significantly affected the T2*-oxygenation-ratio of the striatum (K-W test, p<0.001, Figure 5D). Under isoflurane or sevoflurane, T2*-oxygenation-ratio of the striatum was higher than with propofol (M-W post hoc, p = 0.001 and p<0.001 respectively), midazolam (M-W post hoc, p = 0.016 and 0.001 respectively), medetomidine (M-W post hoc, p = 0.029 and 0.003 respectively) and ketamine-xylazine (M-W post hoc, p = 0.001 and p<0.001 respectively). No statistical differences were observed between propofol, midazolam, medetomidine and ketamine-zylazine for the T2*-oxygenation-ratio.

## Discussion

We demonstrate that ultra-high field MRI reliably estimates the changes in cerebral blood oxygenation induced by anesthetic agents across brain regions.

### Correlation between T2*-oxygenation-ratio and cerebral blood oxygenation

By acting as an endogenous contrast, deoxyhemoglobin allows for *in vivo* noninvasive assessment of CBO without any need for the administration of a contrast agent. The paramagnetic properties of deoxyhemoglobin causes a nonuniform magnetic field, resulting in a rapid dephasing of proton spins on T2*-weighted images [12], [13]. As a result, when oxygenation level increases, the deoxyhemoglobin decreases, the coherence in spin increases, leading to an increase in T2* MR signal within blood vessels, which manifests as lower contrast between blood vessels and brain parenchyma [11], and a higher T2*-oxygenation-ratio [6]. This is consistent with our results. For example, in our study, isoflurane had higher T2*-oxygenation-ratio at 17.2T than propofol whatever the brain region, which is coherent with literature showing higher CBO with isoflurane than with propofol [22], [23]. Deoxyhemoglobin causes not only a reduction in T2* weighted images, but also a phase difference between the vessels and parenchyma [24], [25] that allows to assess blood oxygenation in small vessels [26]. This is in line with our results showing a parallel between T2* magnitude and T2* phase contrast (Figure 2). The increase of the T2* MR signal of the sagittal sinus in our study is indicative of higher blood oxygenation with volatile agents compared to intravenous agents, through a probable increase in CBF [11].

### Effects of anesthetic agents on cerebral blood oxygenation

Having established the reliability and consistency of our CBO ratio, we hypothesized that this measure could be used to assess the effects of anesthetics on CBO. Cerebral blood volume, CBF and CBO, can be influenced by several pharmacological agents that modify deoxyhemoglobin level, and consequently T2* MR signal, even in the absence of a stimulus driven variation of the neuronal activity [27]. In our study we found a striking difference between classical anesthetic categories: volatile anesthetic agents showed a higher T2*-oxygenation-ratio than intravenous anesthetic agents, with low image contrast between brain vessels and parenchyma, due to lower deoxyhemoglobin (i.e. higher oxygenated blood) in the vessels of all studied brain areas. It is established that CBO is affected by the interplay between oxygen consumption and blood flow [11], [28].

How do these findings relate to literature? Anesthetic agents are known to affect cerebral circulation [29], [30]. In literature, although it is established that volatile anesthetics (isoflurane, sevoflurane) induce vasodilation of cerebral arteries [29], there are contradictory reports about their effects on CBF: minimal effects on CBF [31], increase of CBF [32] or even decrease of CBF [33]. These contradictory results are related to the applied minimum alveolar concentration (MAC). It has been demonstrated that for a MAC>1, isoflurane increases CBF [34]. Most intravenous anesthetics are known to reduce CBF. Propofol and midazolam reduce CBF similarly via a decrease in the cerebral metabolic rate of oxygen [1], [35]. Dexmedetomidine decreases CBF, via a direct α_2_-receptor effect of cerebral smooth muscle vasoconstriction and/or a decrease in the cerebral metabolic rate [30]. Xylazine, added to ketamine, decreases CBF (25–65%) [36]. This is coherent with our findings. In fact, our data show a hypersignal of the sagittal sinus under volatile anesthesia (Figure 2), suggesting a higher CBF under isoflurane and sevoflurane compared to intravenous anesthetics [11]. However, our results do not address the question of whether volatile anesthetics change CBF compared to the awake state. In fact, it is very challenging to achieve MRI acquisitions in awake rodents, especially at ultra-high field conditions where animal movements would create huge MR signal artifacts. Still, we may hypothesize that, in our study, while intravenous anesthetics clearly decreased CBF, volatile anesthetics at the applied MAC had a relatively small effect.

So how can we explain the difference in T2*-oxygenation-ratio? Cerebral oxygenation also depends on cerebral metabolism. Anesthetic agents are also known to affect cerebral metabolism [2], [14]. Isoflurane decreases regional metabolic rate of glucose utilization (CMRglu) [2] and CMRO_2_ [37] in a fairly uniform manner in all brain areas. Under 1 MAC sevoflurane anesthesia, metabolism is decreased more than CBF [38]. When sevoflurane anesthesia is deepened from 0.7 to 1.3 MAC, CBF increases, whereas metabolism remains unchanged [39]. Under sevoflurane, CBF is in excess relative to the cerebral oxygen demand [40]. Under propofol, CBF decreases more than CMRO_2_, resulting in a decrease of the CBF/CMRO_2_ ratio [41]. Whole brain CMRglu is decreased under propofol [14], with regional variability in its metabolic effects [14]. Propofol decreases jugular venous oxygen saturation (SjvO_2_), reflecting global cerebral oxygenation, and increases cerebral oxygen extraction rate compared to sevoflurane, which increases SjvO_2_ and decreases cerebral oxygen extraction rate [42]. Under propofol, hemoglobin saturation and jugular bulb venous oxygen partial pressure are lower than under isoflurane [23]. Ketamine causes both increase and decrease in glucose utilization: limbic system and basal ganglia have large increase in glucose utilization and in the somatosensory and auditory systems the glucose metabolism decreases [43]. Ketamine/xylazine decreases cortical tissue oxygen compared to isoflurane in rodents [36].

How can our T2*-oxygenation-ratio be compared to this literature, since T2* does not directly assess CBF nor CMRO_2_? The answer could come from the CBF/CMRO_2_ ratio.

When anesthetics are administered, there is a striking difference between intravenous agents which decrease CBF and CMRO_2_ in a parallel manner, and volatile agents, which affect CMRO_2_ to a larger extent than CBF, because of their intrinsic vasodilatory property [34], [44], [45]. Volatile anesthetics have an increased CBF/CMRO_2_ ratio compared to intravenous agents at rest without any stimulation, consistent with the literature suggesting that the CBF/CMRO_2_ ratio increases or remains unchanged with isoflurane and decreases with propofol [22]. One explanation of our results is that T2* MR signal reflects the CBF/CMRO_2_ ratio. In fact, according to Fick's principle [46]: CMRO_2_ = CBF.C_blood_.(Y_a_-Y_v_); where Y_a_ and Y_v_ are the oxygenation levels of arterial and venous blood (representing the fraction of hemoglobin in the forms of oxyhemoglobin) and C_blood_ is the blood oxygen carrying capacity [28]. Thus CBF/CMRO_2_ = 1/(C_blood_.(Y_a_-Y_v_)). With a conserved blood oxygen carrying capacity, a decrease in deoxyhemoglobin (which manifests as an increase in T2* MR signal) leads to the decrease of the (Y_a_-Y_v_) equation term (because of the increase in venous oxygenation [28] with a constant Y_a_), and to higher CBF/CMRO_2_ ratio. This is exactly what happens under volatile anesthetics according both to our findings and the literature. Thus our T2*-oxygenation-ratio may represent an approximation of the CBF/CMRO_2_ ratio across the brain.

### Implications for functional MRI under anesthesia

As brain blood oxygenation is directly involved in the generation of functional MRI (fMRI) signal, the current results will directly impact the design and interpretation of fMRI investigations under general anesthesia. Because of the uncoupling between CBF and CMRO_2_, volatile anesthetics might interfere with the BOLD genesis during fMRI studies leading to potential confounds. For example, it has been reported that inhalational anesthetics decrease the contrast between the brain and vessels, and need an adaptation of the applied stimuli [47]. Propofol, medetomidine and ketamine-xylazine display a higher T2* contrast across cortical and subcortical brain structures, and are probably more suitable for fMRI studies under general anesthesia [48], [49]. UHF-MRI may be useful to screen and optimize anesthetic drugs use for fMRI studies. Because UHF-MRI is able to monitor the effects of anesthetic agents on the brain *in vivo* in rats, we propose the use of this approach in translational studies during the development of new anesthetic agents.

### Toward a classification for anesthetic agents based on ultra-high field T2* MRI

**Figure 6:**
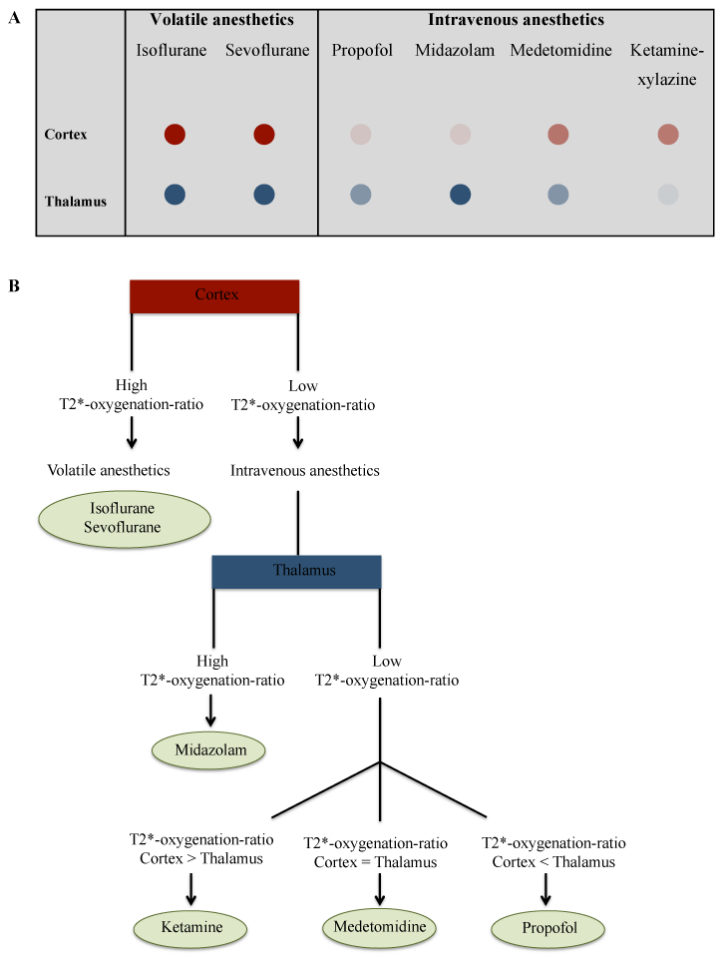
Classification of sedative agents based on their effects on the blood oxygenation level assessed by MRI at 17.2 T. A: The relative oxygenation ratio of isoflurane, sevoflurane, propofol, midazolam, medetomidine and ketamine-xylazine is displayed using a colored disk with variable intensity. Lower color saturation intensity corresponds to lower CBO level, and vice a versa. B: Proposed algorithm to identify anesthetic agents based on relative CBO in the cortex and thalamus as assessed by T2*-oxygenation-ratio. Low signal, T2*-oxygenation-ratio<50000; High signal, T2*-oxygenation-ratio>50000.

Based on the number of statistical differences pairwise and the level of significance, the distribution of the T2*-oxygenation-ratio of cortex had the most striking differences, i.e. the highest discriminative potential, between volatile and intravenous anesthetics. Whereas the T2*-oxygenation-ratio of the thalamus had the most striking differences among intravenous anesthetics (Figure 5). Thus we could classify the different anesthetic agents based on their T2*-oxygenation-ratio in cortex and thalamus (Figure 6A). We suggest an algorithm based classifier that identifies the anesthetic agent based on its T2*-oxygenation-ratio at 17.2T (Figure 6B). The first and second algorithm nodes compare the T2* index to a threshold of 50000, which was identified from this study data (Figure 5). Thus, starting from the anesthetic agents effects on cerebral blood oxygenation, we suggest that ultra-high field T2*-oxygenation-ratio of the cortex and the thalamus can establish a drug profile and a drug classification for anesthetics. Future work could validate the application of decoding approaches and machine learning algorithms to identify a sedative drug from its MRI signal.

## Supporting Information

Figure S1 Normality of T2*-oxygenation-ratio.
(PDF)

Dataset S1 **Data used to construct** **Figures 3** **,** **4** **,** **5** **.**
(DOCX)
