# Supplementary material for: Sedation Agents Differentially Modulate Cortical and Subcortical Blood Oxygenation: Evidence from Ultra-High Field MRI at 17.2 T
Source: PLoS One. 2014 Jul 22;9(7):e100323. doi: 10.1371/journal.pone.0100323 (PMC4106755; doi:10.1371/journal.pone.0100323)

Supporting Information Figure S1: Normality of T2*-oxygenation-ratio measured during Isoflurane sessions

A Cortex

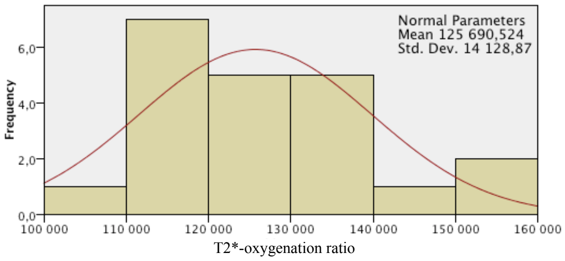

B Hippocampus

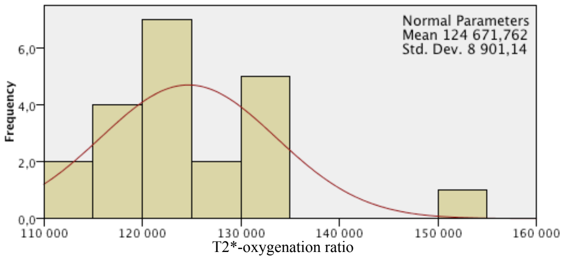

C Striatum

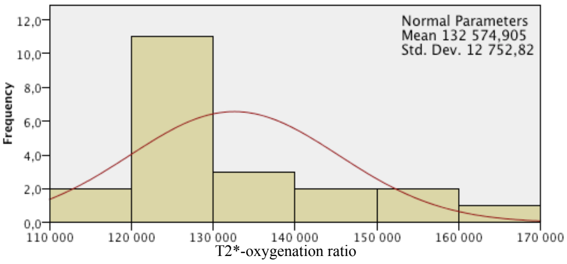

D Thalamus

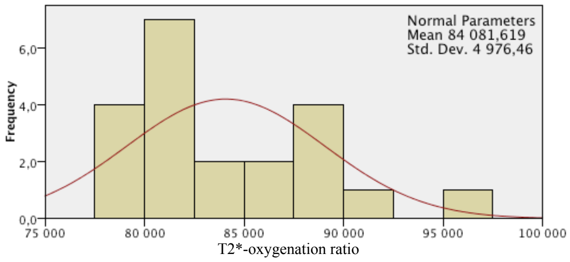

Supplement: Figure S1 — Normality of T2*-oxygenation-ratio. (PDF) [file pone.0100323.s001.pdf]
