# Supplementary material for: Sedation Agents Differentially Modulate Cortical and Subcortical Blood Oxygenation: Evidence from Ultra-High Field MRI at 17.2 T
Source: PLoS One. 2014 Jul 22;9(7):e100323. doi: 10.1371/journal.pone.0100323 (PMC4106755; doi:10.1371/journal.pone.0100323)
Supplement: Dataset S1 — Data used to construct Figures 3 , 4 , 5 . (DOCX) [file pone.0100323.s002.docx]

|  | **Isoflurane T2* Oxygenation Ratio** | | |  |
| --- | --- | --- | --- | --- |
| **Animal** | **Cortex** | **Striatum** | **Thalamus** | **Hippocampus** |
| 1 | 130447 | 127422 | 90097 | 130059 |
| 2 | 121137 | 156716 | 88674 | 118400 |
| 3 | 134203 | 118952 | 83276 | 117131 |
| 4 | 124744 | 124849 | 78845 | 112642 |
| 5 | 122637 | 129341 | 89002 | 132618 |
| 6 | 154701 | 157751 | 86065 | 152285 |
| 7 | 121096 | 121329 | 81501 | 126182 |
| 8 | 113201 | 129892 | 80758 | 120068 |
| 9 | 103477 | 114872 | 80758 | 123460 |
| 10 | 111953 | 129892 | 89635 | 122747 |
| 11 | 113356 | 123016 | 82254 | 114127 |
| 12 | 118113 | 127672 | 86022 | 124737 |
| 13 | 141513 | 140046 | 78153 | 115718 |
| 14 | 133598 | 131338 | 84128 | 131422 |
| 15 | 127372 | 162935 | 79027 | 115579 |
| 16 | 132226 | 139993 | 81232 | 133724 |
| 17 | 156954 | 125305 | 81232 | 123583 |
| 18 | 111890 | 124265 | 89413 | 126737 |
| 19 | 114955 | 128651 | 77609 | 131439 |
| 20 | 113427 | 135843 | 81298 | 122776 |
| 21 | 138501 | 133993 | 96735 | 122673 |
|  |  |  |  |  |
|  |  |  |  |  |
|  | **Sevoflurane T2* Oxygenation Ratio** | | |  |
| **Animal** | **Cortex** | **Striatum** | **Thalamus** | **Hippocampus** |
| 1 | 118082 | 137394 | 112162 | 69301 |
| 2 | 168291 | 161563 | 98091 | 97862 |
| 3 | 210252 | 330152 | 268108 | 223365 |
| 4 | 171417 | 146813 | 89742 | 145069 |
| 5 | 85973 | 149050 | 81149 | 86804 |
|  |  |  |  |  |
|  |  |  |  |  |
|  | **Propofol T2* Oxygenation Ratio** | | |  |
| **Animal** | **Cortex** | **Striatum** | **Thalamus** | **Hippocampus** |
| 1 | 13799 | 28708 | 28320 | 19265 |
| 2 | 13775 | 22782 | 26512 | 13264 |
| 3 | 7077 | 16792 | 19754 | 9128 |
| 4 | 23416 | 27947 | 31835 | 15967 |
| 5 | 17123 | 29323 | 25024 | 12550 |
|  |  |  |  |  |
|  |  |  |  |  |
|  | **Midazolam T2* Oxygenation Ratio** | | |  |
| **Animal** | **Cortex** | **Striatum** | **Thalamus** | **Hippocampus** |
| 1 | 9345 | 26897 | 81645 | 12418 |
| 2 | 26138 | 51487 | 82171 | 24488 |
| 3 | 22107 | 91442 | 50089 | 19858 |
| 4 | 33763 | 91937 | 96674 | 47969 |
| 5 | 13941 | 57507 | 47281 | 19986 |
|  |  |  |  |  |
|  |  |  |  |  |
|  | **Medetomidine T2* Oxygenation Ratio** | | |  |
| **Animal** | **Cortex** | **Striatum** | **Thalamus** | **Hippocampus** |
| 1 | 18367 | 31894 | 19247 | 24943 |
| 2 | 39385 | 33169 | 21493 | 22233 |
| 3 | 32925 | 51239 | 36835 | 24634 |
|  |  |  |  |  |
|  |  |  |  |  |
|  | **Ketamine/Xylazine T2* Oxygenation Ratio** | | | |
| **Animal** | **Cortex** | **Striatum** | **Thalamus** | **Hippocampus** |
| 1 | 28023 | 15667 | 7137 | 12084 |
| 2 | 25784 | 8828 | 6472 | 9742 |
| 3 | 17987 | 9748 | 8584 | 10824 |
